# Packed bed reactor for degradation of simulated cyanide-containing wastewater

**DOI:** 10.1007/s13205-014-0261-6

**Published:** 2014-10-26

**Authors:** Virender Kumar, Vijay Kumar, Tek Chand Bhalla

**Affiliations:** Department of Biotechnology, Himachal Pradesh University, Summer Hill, Shimla, 05 HP India

**Keywords:** *Serratia marcescens* RL2b, Packed bed reactor, Cyanide, Retention time

## Abstract

The discharge of cyanide-containing effluents into the environment contaminates water bodies and soil. Effective methods of treatment which can detoxify cyanide are the need of the hour. The aim of the present study is to develop a bioreactor for complete degradation of cyanide using immobilized cells of *Serratia marcescens* RL2b. Alginate-entrapped cells of *S. marcescens* RL2b were used for complete degradation of cyanide in a packed bed reactor (PBR). Cells grown in minimal salt medium (pH 6.0) were harvested after 20 h and exhibited 0.4 U mg^−1^ dcw activity and 99 % cyanide degradation in 10 h. These resting cells were entrapped using 3 % alginate beads and packed in a column reactor (20 × 1.7 cm). Simulated cyanide (12 mmol l^−1^)-containing wastewater was loaded and fractions were collected after different time intervals at various flow rates. Complete degradation of 12 m mmol l^−1^ (780 mg l^−1^) cyanide in 10 h was observed at a flow rate of 1.5 ml h^−1^. The degradation of cyanide in PBR showed direct dependence on retention time. The retention time of cyanide in the reactor was 9.27 h. The PBR can degrade 1.2 g of cyanide completely in 1 day.

## Introduction

Cyanide is considered to be among the first nitrogen-containing carbon molecules formed during chemical evolution in the pre-biotic era on the Earth. Cyanide compounds are widely distributed in biotic and abiotic components of ecosystems. A large number of plants and microorganisms have compounds capable of producing cyanides (Knowles 1976). Some agriculturally important plants such as almond, alfalfa, bamboo, cassava, cotton, corn, cherry, flex, peach, plum, potato and sorghum are well known for their cyanogenic nature (Dwivedi et al. 2011).

Cyanide compounds are extensively used in acrylic fibers and resin production industries, coal industries, cassava starch production industries, plastic production and mining industries (Hamel 2011; Potivichayanon and Kitleartpornpairoat 2010). Approximately, 1.1 million metric tons of cyanide is produced annually worldwide as estimated by the International Cyanide Management Institute (ICMI). The discharge of cyanide-containing compounds and complexes into the environment as industrial waste from these industries results in contamination of soil water and air with compounds such as hydrogen cyanide, thiocyanate and cyanate. The reaction of cyanide with metals and heavy metals results in the formation of metal cyanide complexes. A number of industrial effluents contain cyanide at a concentration exceeding 100 mg l^−1^ and its leaching through soil results in contamination of groundwater (Park et al. 2008).

The toxicity of cyanide has been well documented. It acts as a very strong inhibitor of cytochrome oxidase, one of the very crucial enzymes in mitochondria, and blocks the ultimate transfer of electrons to molecular oxygen in the mitochondrial electron transport chain Luque-Almagro et al. (2011). It is thus inevitable to degrade cyanide in industrial effluents or remediate contaminated soil and water to reduce its level to the permissible limit of 0.2 mg l^−1^ in effluents. A number of cyanide treatment methods based on physiochemical processes are in use, including copper-catalyzed chemical process, hydrogen peroxide process, alkaline chlorination and SO_2_/air remediation process (Kao et al. 2004). High operational cost vis-à-vis generations of chemical wastes are limitations of these processes.

Microbial detoxification of cyanide has emerged as an inexpensive, eco-friendly and viable alternative to physiochemical processes for the treatment of cyanide in industrial effluents or contaminated sites (Huertas et al. 2010). The microorganisms reported to degrade cyanide include *Bacillus* sp. (Wu et al. 2013), *Pseudomonas fluorescens* (Dursun et al. 2009), *Fusarium solani*, *Trichoderma polysporum*, *Penicillium miczynski*, *Azotobacter vinelandii *(Dash et al. 2009a, b). Among these, *Azotobacter vinelandii* degrades 69 % of 4 mmol l^-1^ sodium cyanide while *Bacillus* sp CN-22 has been reported to effectively degrade cyanide upto 200 mgl^-1^ concentration (Wu et al. 2013).These organisms have been used in free cells for cyanide detoxification studies. Recently, *S. marcescens* RL2b has been isolated and reported as an efficient cyanide-degrading bacterium from our laboratory which actively degraded 12 mmol l^−1^ potassium cyanide (Kumar et al. 2013). This organism is one of the most efficient cyanide-degrading organisms reported so far. In the present communication, degradation of cyanide by alginate-entrapped cells of *S. marcescens* RL2b in a packed bed reactor has been reported.

## Methods

### Chemicals

Analytical-grade chemicals were purchased from SD Fine Chemical media. The medium components were procured from Himedia Ltd, Mumbai, India.

### Culture of *Serratia marcescens* RL2b

The *S. marcescens* RL2b used in the present study was isolated previously at the Department of Biotechnology, Himachal Pradesh University, Shimla, India (Kumar et al. 2013).

## Optimization of harvesting time of *S. marcescens* RL2b resting cells

The *S. marcescens* RL2b was grown in minimal media (2.5 g K_2_HPO_4_; 2 g KH_2_PO_4_; 0.5 g; 32 mg FeSO_4_; 64 mg CaCl_2_ and 1 % glycerol per liter of media) at 30 °C temperature, pH 6.0, and tested for the ability to degrade cyanide by harvesting culture at different time intervals (12, 16, 20 and 24 h). The cells were harvested at 6,000 g and 4 °C temperature and suspended in 0.1 mol l^−1^ K_2_HPO_4_/KH_2_PO_4_ buffer (pH 6.0). The resting cells were fed with 12 mM cyanide. The cyanide degradation activity (U mg^−1^ dcw) where one unit of degradation activity is defined as micromoles of cyanide degraded per minute per milligram dry cell weight and percent cyanide removal was measured.

## Degradation of cyanide by resting cells

The *S. marcescens* RL2b cells harvested after 20 h showed maximum cyanide degradation activity. These resting cells were washed twice with phosphate buffer and then used for complete degradation of cyanide. Two ml reaction mixture was prepared containing 400 µl cells (4.6 mg ml^−1^ dcw) and 25 µl cyanide (12 mmol l^−1^) and 1,575 µl of 0.1 mol l^−1^ K_2_HPO_4_/KH_2_PO_4_ buffer (pH 6.0). Reaction was carried at 35 °C. The cyanide level was monitored after every hour till complete degradation using picric acid assay (Fisher and Brown 1952).

## Immobilization of *S. marcescens* RL2b cells

### Selection of buffer

Three buffer systems, i.e., 0.1 mol l^−1^ citrate buffer (pH 4.0, 5.0, 6.0, 7.0), 0.1 mol l^−1^ borate buffer (pH 8.25, 9.0, 9.5 and 10.0) and 0.1 mol l^−1^ Tris–HCl buffer, were used. The reaction was performed at 35 °C reaction conditions. The buffer system and pH in which maximum cyanide degradation was observed were selected for entrapment in subsequent experiments.

### Alginate concentration

The resting cells of *S. marcescens* RL2b (4.6 mg ml^−1^ dcw) were suspended in alginate concentration varying from 1 to 4 %. Beads were prepared in 0.2 mol l^−1^ calcium chloride and stored in 0.1 mol l^−1^ citrate buffer. The alginate concentration at which maximum degradation of cyanide was observed was selected for immobilization of cells. The cells of *S. marcescens* RL2b were immobilized in 3 % alginate beads and cyanide degradation was monitored at various intervals of time.

### Degradation of cyanide in packed bed reactor

A packed bed reactor (PBR) using alginate-entrapped cells of *S. marcescens RL2b* was designed for bioremediation of simulated cyanide-containing effluents. The entrapped cells were packed in a column of 20 cm length and a diameter of 1.7 cm. Immobilized beads were packed up to a length of 15 cm and washed with 0.1 mol l^−1^ citrate buffer (pH 6.0). The simulated effluent containing 12 mmol l^−1^ potassium cyanide solution was eluted through the reactor. The cyanide degradation was monitored at different flow rates of the simulated cyanide-containing effluent through the reactor. The cyanide concentration in the fractions was estimated.

### Retention time

Retention time of simulated cyanide water effluent in PBR was calculated using the formula *τ* = *V*/*F* where *V* is the volume of PBR (ml) and *F* is the flow rate (ml h^−1^). The equation was further extended to calculate per day degradation efficiency of the PBR as follows: *F*(*S*_i_ − *S*_f_)24, where *F* is flow rate, *S*_i_ is the initial substrate concentration and *S*_f_ is the final substrate concentration.

### Assay method

Samples were withdrawn in small aliquots at different intervals of time, centrifuged and the cyanide level in the supernatant was assayed using picric acid assay method (Fisher and Brown 1952). Absorbance was measured at 520 nm and the concentration of cyanide was calculated from the standard curve of cyanide estimation (prepared by assaying KCN ranging from 1.5 to 13.5 µmol l^−1^).

## Results

### Optimization of harvesting time

**Fig. 1:**
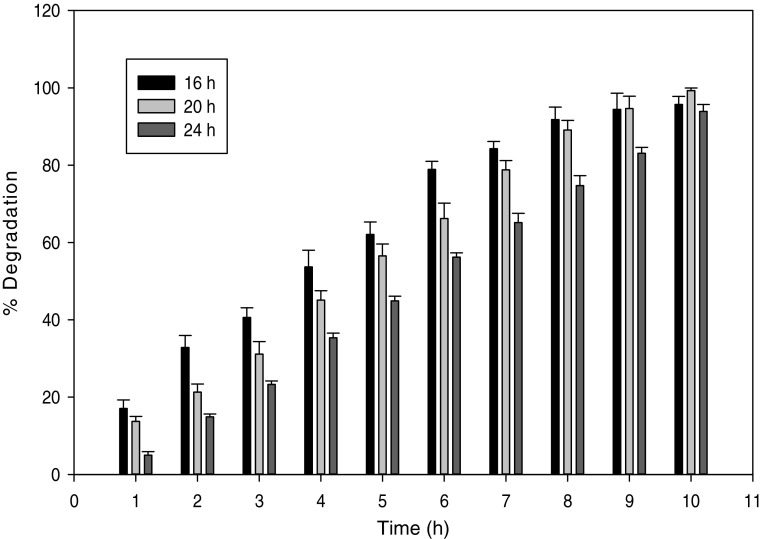
Percent cyanide degradation vs harvesting time for preparation of resting cells of *S. marcescens* RL2b. All points are the means of triplicate reactions, and *error bar* indicates the standard deviation

In the present investigation, cells harvested after 20 h exhibited maximum cyanide degradation activity (0.4 U mg^−1^ dcw). The cells harvested after 16 and 24 h showed less degradation activity, i.e., 0.28 and 0.3 U mg^−1^ dcw. Complete degradation of 12 mmol l^−1^ cyanide was achieved in approximately 10 h with the resting cells harvested after 20 h (Fig. 1).

### Degradation of cyanide by alginate-entrapped cells

Five different matrices were used in the present study. Based on the shape, preparation complexity, mechanical strength and immobilization efficiency, the alginate was selected. Citrate buffer (pH 6.0) was used for immobilized cells as the cells retained higher degradation activity (0.5 U mg^−1^ dcw) in this buffer as compared to borate (0.4 U mg^−1^ dcw) and Tris–HCl buffer (0.37 U mg^−1^ dcw). Using RL2b cells (4.6 mg ml^−1^ dcw), the alginate concentration for the preparation of beads was optimized. The cells entrapped in 1 and 2 % sodium alginate demonstrated similar degradation. The highest degradation of cyanide was observed in cells entrapped in 3 % alginate beads (2 mm). The beads were recovered and reused for cyanide degradation three times, after which the degradation activity declined rapidly.

### Packed bed reactor (PBR)

**Fig. 2:**
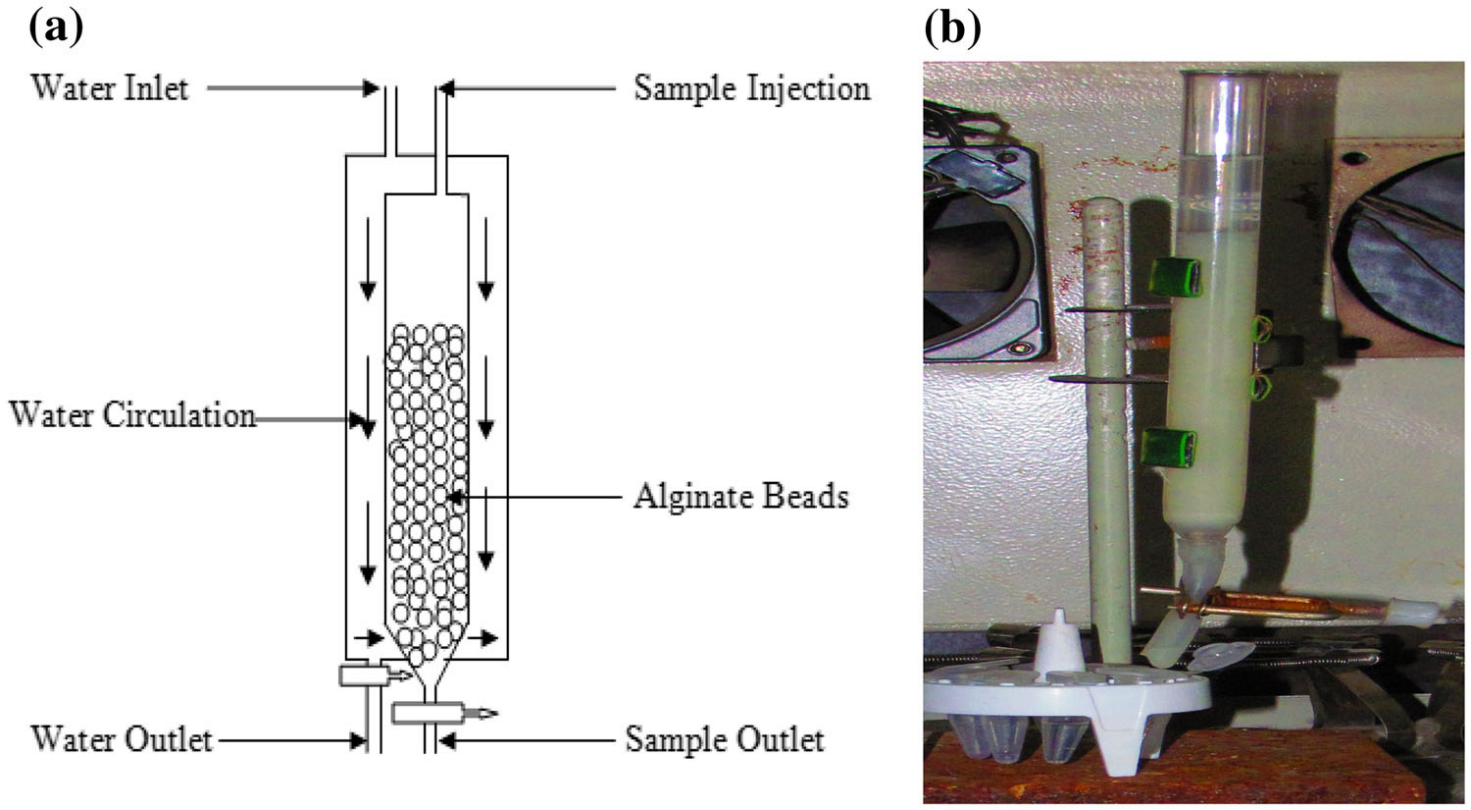
**a** Schematic diagram of packed bed bioreactor for cyanide degradation by alginate-immobilized *S. marcescens* RL2b cells. **b** Packed bed reactor in operation

**Fig. 3:**
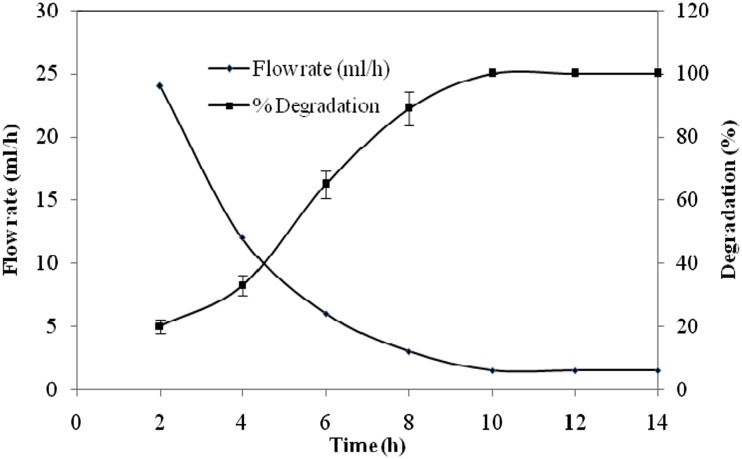
Effect of flow rate on cyanide degradation in packed bed bioreactor by alginate-immobilized *S. marcescens* RL2b cells. All points are the means of triplicate reactions, and *error bar* indicates the standard deviation

In the present investigation, alginate-entrapped cells of *S. marcescens* RL2b were used in a packed bed reactor. The flow rate was adjusted to 1.5 ml h^−1^, at which complete degradation of 12 mmol l^−1^ (780 mg l^−1^) cyanide in 10 h was observed. A design of the immobilized packed bed reactor is shown in Fig. 2. The correlation of cyanide degradation and retention time is depicted in Fig. 3. It was seen that degradation of cyanide by the alginate-entrapped cells depends on the retention time of the cyanide in the reactor. Increase in retention time increased the quantum of cyanide degradation.

### Retention time

The flow rate of the PBR was optimized to 1.5 ml h^−1^ and the volume of the reactor was 13.9 cm^3^ (ml). The optimum retention time was calculated to be 9.27 h. If the PBR is operated under these conditions, it will degrade 1.20 g cyanide in 1 day with 99 % degradation and without much decrease in the rate of cyanide degradation by immobilized cells, where *S*_i_ and *S*_f_ are 780 and 0.78 mg l^−1^, respectively.

## Discussion

The enzymes responsible for degradation are produced at a specific time during the growth cycle. It is necessary to accurately estimate the time of enzyme production. In the present study, cells harvested after 20 h possessed 0.4 U mg^−1^ dcw cyanide degradation activity, possibly due to the large amount of enzyme capable of degradation produced by the cells during this phase. The resting cells harvested after 20 h completely degraded 12 mmol l^−1^ cyanide in approximately 10 h. The degradation of toxic compounds by free cells has many disadvantages such as inhibition at high concentration, recalcitrance, difficulty in recovery from reactions and loss of activity. The immobilization on suitable matrices leads to harness the maximum potential of microbial cells to remove such toxic compounds without substrate inhibition and with easy recovery and most importantly reusability of cells. There are reports on simultaneous adsorption and degradation of zinc and iron cyanides by *Rhizopus oryzae* (MTCC 2541). Granular activated carbon was used for cell immobilization (Dash et al. 2009a, b, 2010; Zhou et al. 2007). Sodium alginate-immobilized cells of fungus *Trichoderma koningii* were used to degrade cyanide and ferrocyanide (Campos et al. 2006). In the present study, matrices used were characterized for their shape, preparation complexity and immobilization efficiency before selection. Based on these findings, alginate was selected. The cells showed very good degradation activity (0.5 U mg^−1^ dcw) in citrate buffer than in borate and Tris–HCl buffer, i.e., 0.4 and 0.37 U mg^−1^ dcw, respectively, so citrate buffer (pH 6.0) was used for immobilized cells The concentration of alginate for the preparation of beads was optimized using RL2b cells (4.6 mg ml^−1^ dcw). Maximum cyanide degradation was observed in cells entrapped in 3 % alginate beads (2 mm). The beads of immobilized cells were recovered and reused for cyanide degradation three times.

Bioreactors can be potentially useful for bioremediation purposes, as they offer low energy consumption and high removal efficiency. Immobilization can be coupled to enhance stability and reusability of cells. The objective of the development of such a biodegradation system is to reduce the toxicity of cyanide-containing industrial effluents. Simultaneous biodegradation of cyanide and formamide by immobilized *Fusarium oxysporum* (CCMI 876) and *Methylobacterium* sp. (CCMI 908) in a packed bed reactor was reported (Sirianuntapiboon and Chuamkaew 2007). Packed cage rotating biological contactor containing the highest cyanide concentration of 40 mg l^−1^ showed almost constant cyanide removal efficiencies of 89 and 90 %, even when the system was controlled under the lowest retention time of 8 h (Maniyam et al. 2011; Mirizadeh et al. 2014). A packed bed reactor was developed in the present study using alginate-entrapped cells of *S. marcescens* RL2b. When the flow rate was set to 1.5 ml h^−1^, complete degradation of 12 mmol l^−1^ (780 mg l^−1^) cyanide in 10 h was observed, i.e. 100 % removal of cyanide. The extent of cyanide degradation by the alginate-entrapped cells depends on the retention time of the cyanide in the reactor. Increase in retention time increased the quantum of cyanide degradation (Fig. 3). The alginate-entrapped cells of *S. marcescens* RL2b may prove to be a better alternative for degradation of cyanide-contaminated wastewater. The bioreactor developed in the present study can be scaled up and employed for remediation of cyanide-contaminated water at larger scale.
